# Long-term low-dose acetylsalicylic use shows protective potential for the development of both vascular dementia and Alzheimer’s disease in patients with coronary heart disease but not in other individuals from the general population: results from two large cohort studies

**DOI:** 10.1186/s13195-022-01017-4

**Published:** 2022-05-28

**Authors:** Thi Ngoc Mai Nguyen, Li-Ju Chen, Kira Trares, Hannah Stocker, Bernd Holleczek, Konrad Beyreuther, Hermann Brenner, Ben Schöttker

**Affiliations:** 1 Division of Clinical Epidemiology and Aging Research, German Cancer Research Center (DKFZ), 69120 Heidelberg, Germany; 2 Network Aging Research, University of Heidelberg, 69115 Heidelberg, Germany; 3 Saarland Cancer Registry, 66119 Saarbrücken, Germany

**Keywords:** Acetylsalicylic acid, Aspirin, Dementia, Coronary heart disease, Alzheimer’s disease, Cohort study, Meta-analysis, Prevention

## Abstract

**Background:**

No population-based cohort study investigated a potential inverse association between long-term low-dose acetylsalicylic acid (ASA) use and all-cause dementia and its two most common sub-types Alzheimer’s disease (AD) and vascular dementia (VD) so far.

**Methods:**

Cox regression models with inverse probability of treatment weighting to model the underlying cardiovascular risk were used to assess the associations of low-dose ASA use with all-cause dementia, AD, and VD incidence in community-dwelling older adults from the German ESTHER study (*N *= 5258) and the UK Biobank (*N *= 305,394). Inclusion criteria were age of 55 years or older and completed drug assessment. Meta-analyses of the individual participant data from the two prospective cohort studies were performed.

**Results:**

Four hundred seventy-six cases of all-cause dementia, 157 cases of AD, and 183 cases of VD were diagnosed over a median of 14.3 years of follow-up in ESTHER. In the UK Biobank, 5584 participants were diagnosed with all-cause dementia, 2029 with AD, and 1437 with VD over a median of 11.6 years. The meta-analysis of both cohorts revealed a weak reduction in hazards for all-cause dementia (hazard ratio (HR) [95% confidence interval (CI)]: 0.96 [0.93 to 0.99]). The strongest protective effect of low-dose ASA was observed in participants with coronary heart disease (CHD) in both cohorts, and a significant interaction was detected. In particular, in meta-analysis, a 31% reduction in hazard for AD, 69% for VD and 34% for all-cause dementia were observed (HR [95% CI]: 0.69 [0.59 to 0.80], 0.31 [0.27 to 0.35], 0.46 [0.42 to 0.50], respectively). Furthermore, compared to non-users, users of low-dose ASA for 10 years or longer (who likely use it because they have CHD or a related diagnosis putting them at an increased risk for cardiovascular events) demonstrated a strong protective effect on all dementia outcomes, especially for VD (HR [95% CI]: 0.48 [0.42 to 0.56]) whereas no protective associations were observed with shorter low-dose ASA use.

**Conclusions:**

The protective potential of low-dose ASA for all-cause dementia, AD, and VD seems to strongly depend on pre-existing CHD and the willingness of patients to take it for a minimum of ten years.

**Supplementary Information:**

The online version contains supplementary material available at 10.1186/s13195-022-01017-4.

## Introduction

For decades, the non-steroidal anti-inflammatory drug (NSAID) acetylsalicylic acid (ASA) has been widely used at a low dose of 100 to 300 mg per day for secondary prevention of atherosclerotic cardiovascular disease (ASCVD) [1]. Although the risks and benefits of ASA are well known, its potential for neuroprotection is still a matter of debate. Through its anti-inflammatory property, ASA could potentially prevent or delay the onset of Alzheimer's disease (AD) [2–5]. Moreover, as an anti-thrombotic agent, ASA further helps to reduce cerebrovascular disease, which may also contribute to vascular dementia (VD) prevention [6].

Systematic reviews and meta-analyses of observational studies but not randomized controlled trials (RCTs) supported the possibility that low-dose ASA could protect against dementia [7, 8]. However, no population-based, observational cohort study has been performed so far. The two RCTs published so far excluded subjects with CVD, which are the main users of low-dose ASA in clinical routine, included rather old study populations (median ages of 65 and over 70 years) and had short median treatment durations of 4.4 and 4.7 years [7]. Studies with rather young study populations and long-term follow-up are especially needed because it has been observed that NSAID use might only protect against AD when initiated long before cognitive decline begins [9]. Therefore, despite available data from RCTs, there is still a need for a population-based cohort study with long-term follow-up excluding adults aged 80 and older. This study investigated the association of low-dose ASA with all-cause dementia, AD, and VD incidence using data from two large, population-based cohorts with more than 10 years of follow-up.

## Methods

### Study design and population

We used data from two prospective cohorts: the ESTHER study from Germany and the UK Biobank from the United Kingdom (UK). ESTHER (full German name: *Epidemiologische Studie zu Chancen der Verhütung, Früherkennung und optimierten Therapie chronischer Erkrankungen in der älteren Bevölkerung*) is an ongoing population-based cohort study whose details have been reported elsewhere [10]. In brief, 9940 individuals aged 50–75 years were recruited via their general practitioners (GPs) during a routine health check-up between July 2000 and December 2002. After 2, 5, 8, 11, 14, and 17 years, participants and their GPs were contacted again and asked to complete questionnaires on health status, medical diagnoses, and treatments. For this project, we included participants who had a drug assessment at either baseline or 2-year follow-up. After excluding those aged <55 years, with missing dementia diagnosis information from their GPs, or who developed dementia between baseline and the 2-year follow-up, we arrived at *N *= 5258 for analyses (Supplemental Fig. A1).

The UK Biobank is a large-scale, prospective cohort study. Between 2006 and 2010, more than half a million study participants aged 40 to 69 years who lived up to 25 miles from one of 22 study assessment centers in England, Scotland, and Wales were recruited [11]. At baseline assessment visit, participants completed a touch-screen questionnaire, a brief computer-assisted interview, had physical and functional measurements taken and biological samples collected [12]. Follow-up of health-related outcomes was enabled through linkage to routinely available data from the UK National Health Service (e.g., mortality, cancer registrations, hospital admissions, primary care data), and in this analysis, we used the most up to date available data (up to 31 March 2021 for England and Scotland and 28 February 2018 for Wales). From 502,492 participants at baseline, we excluded those aged <55 years and those with missing information on sex, drug assessment, or dementia diagnosis (Supplemental Fig. A2). Overall, 305,394 participants were included.

### Assessment of drugs

In ESTHER, an assessment of low-dose ASA use was made by combining the physicians’ questionnaire at baseline (ASA as a prescription drug) and the participants’ questionnaire at 2-year follow-up (ASA as an over-the-counter drug). A participant was considered a low-dose ASA user when there was a record of ASA use at the dose of ≤ 300 mg per day, either at baseline or at 2-year follow-up. The cohort entry date was set at the date of arrival of the 2-year follow-up questionnaire.

In the UK Biobank, users of low-dose ASA were identified through the list of codes used by clinic nurses to code drugs that study participants brought to the verbal interview [13]. The dosage of drugs was not recorded. However, since short-term used drugs were explicitly excluded from this drug assessment, we assumed that ASA was prescribed at a low dose. We further checked this assumption in UK Biobank participants with primary care prescription data whose dosing information was available. Drugs in the GP prescription data were coded using the Read v2, British National Formulary (BNF) and dm+d [14, 15]. Among 221,734 participants of the UK Biobank with drug information available in both interview data and primary care data, 25,528 (11.5%) received at least one prescription of low-dose ASA prior to the baseline assessment date and 18,454 of these (72.3%) were also identified at the verbal interview. The level of agreement in identifying low-dose ASA users between interview data and primary care data was close to the threshold between moderate and substantial agreement (Cohen’s Kappa coefficient, 0.59, Supplemental Table A1).

### Ascertainment of incident dementia outcomes

In ESTHER, GPs of all participants (including those who had dropped out during follow-up due to illness or death) were asked to provide information on potential dementia diagnoses through standardized questionnaires at the 14- and 17-year follow-up. Diagnoses reported by GPs were confirmed through available medical records of neurologists, psychiatrists, memory, or other specialized clinics [16]. In Germany, diagnosis of AD follows the guidelines of the National Institute on Aging-Alzheimer's Association workgroups [17] or the IWG-2 criteria [18].

In the UK Biobank, incident dementia cases were obtained through algorithmic combinations of linked data from hospital admissions and death registries [19]. In this analysis, we excluded participants who had already been diagnosed with dementia at study entry, either in hospital admission data or self-reported during the baseline interview. In the ESTHER study, mental inability to fill self-administered questionnaires was an exclusion criterion applied by the GPs during recruitment, which practically excluded individuals with dementia from taking part in the baseline assessment of the cohort.

### Assessment of covariates

In ESTHER, study participants completed a standardized, comprehensive, self-administered questionnaire at baseline, providing information on sociodemographic characteristics, medical history, health status, family history of diseases, and lifestyle factors. Their GPs completed a standardized health check-up form and documented current drug prescriptions. At 2-year follow-up, an additional self-reported questionnaire on medication use was sent to participants. Total and high-density lipoprotein (HDL) cholesterol were assessed in blood samples taken at baseline by enzymatic colorimetric tests (analytes Chol2 2100 and HDLC3 450). C-reactive protein (CRP) was determined by immunoturbidimetry (analyte CRPL3), and serum creatinine was measured by the kinetic Jaffé method (analyte CREJ2) on a Cobas 8000 C701 [20]. The apolipoprotein E (*APOE*) epsilon alleles were determined based on the allelic combination of the single nucleotide polymorphisms (SNPs) *rs7412* and *rs429358* using TaqMan SNP genotyping assays with genotypes analyzed in an endpoint allelic discrimination read using a PRISM 7000 Sequence detection system (Applied Biosystems, Foster City, CA) [21].

In the UK Biobank, participants completed a touch-screen questionnaire at the assessment center visit, from which socio-demographics (e.g., education, household number, and income), lifestyle (e.g., smoking status, alcohol consumption, and physical activities), psychosocial factors, mental health, and medical history were obtained [22]. Thereafter, participants interviewed a trained nurse to give further detailed information on major illnesses and disabilities, operations, and regular prescription medication taken. Physical measurements, including blood pressure and anthropometry, and biological samples were also taken [23]. Total cholesterol and serum creatinine were analyzed by enzymatic tests, CRP by immuno-turbidimetric test, and HDL by enzyme immuno-inhibition test utilizing the Beckman Coulter AU5800 system (Beckman Coulter, UK) [24]. Genome-wide genetic data were available for 488,377 participants, of whom 49,950 were genotyped on the UK BiLEVE Axiom array while the remaining were run on the UK Biobank Axiom array [25]. As mentioned for ESTHER, depending on the combination of alleles at *rs429358* and *rs7412* variants, individuals were classified according to one of the six common *APOE* genotypes (ε2ε2, ε2ε3, ε3ε3, ε2ε4, ε3ε4 and ε4ε4).

### Statistical analysis

Cox proportional hazards regression models were used to assess the longitudinal associations of low-dose ASA with all-cause dementia, AD, and VD in comparison with study participants using no low-dose ASA. In a simple model, we adjusted for important risk factors for dementia: age, sex, education, *APOE ε4* genotypes, body mass index (BMI), smoking status, alcohol consumption, physical activity, diabetes, hypertension, coronary heart disease (CHD) and depression. In the main model, we applied the inverse probability of treatment weighting (IPTW) using propensity scores (PS). The results of the logistic regression models used to derive the PS from 57 variables for ESTHER and 47 variables for UK BIOBANK are reported in Supplementary Table A2 and A3. Factors included in the PS are cardiovascular risk or preventive factors (selected based on knowledge of the scientific literature). Weights were assigned to participants based on the inverse of their probability of receiving low-dose ASA, as estimated by the PS. Weights that exceeded the 99th percentiles were set to that threshold [26, 27]. In sensitivity analysis, we also conducted all analyses with PS matching (1:1). The PS matching analysis confirmed our results obtained with IPTW, but resulted in a less precise effect estimation; as only about three-quarters of low-dose ASA users could be matched to one control with a similar propensity to get the drug prescribed (data not shown).

The IPTW analyses were carried out for the total population and stratified by age, sex, CHD status, and *APOE* genotype. Furthermore, interaction tests between stratifying variables and low-dose ASA use were carried out. In a sensitivity analysis, we performed a 5-year lag-time model in which dementia cases in the first 5 years of follow-up were excluded. Finally, we carried out a multivariable logistic regression with restriction to only participants with primary care data available from the UK Biobank because the duration of use was only available from this data source. low-dose ASA users in this analysis were categorized into four groups: non-users, users of ≤5 years, users from 5 to ≤10 years, and users of >10 years, depending on the length of low-dose ASA use from the first prescription identifiable in the primary care data to defined end of follow-up: dementia diagnosis, loss to follow-up, death or censoring date (31 March 2021 for study participants from England and Scotland and 28 February 2018 for study participants from Wales).

All statistical analyses were carried out with SAS v.9.4 (North Carolina, USA). All tests were performed two-sided using an α-level of 0.05. To our knowledge, missing values of covariates were missing at random. Multiple imputation of five data sets was undertaken to deal with missing values, and the results of these five imputed datasets were combined by the SAS procedure PROC MIANALYZE. Five imputed datasets have been suggested to be sufficient to get a reasonably accurate estimate [28]. The proportion of missing values imputed per covariate is shown in Supplemental Table A4. All analyses were first carried out separately in both cohorts and pooled by fixed effects meta-analyses thereafter. Meta-analyses were conducted with the Comprehensive Meta-analysis 2.0 software (Biostat, Englewood, NJ, USA).

## Results

Table 1 shows the baseline characteristics of the included study participants of both cohorts. While the UK Biobank study sample included proportionally more study participants younger than 64 years, the proportion of individuals aged above 70 years was higher in the ESTHER cohort (22.0% vs 0.8%). Both cohorts included 46% males. Presumably, due to the age difference, the ESTHER study participants were less physically active, had more frequently HDL levels <40 mg/dL, CRP ≥3 mg/L, and had a higher prevalence of depression, hypertension, diabetes, and CHD. ESTHER’s study participants also smoked more but consumed alcohol less often, and as opposed to 44.1% in UK Biobank, only 11.6% finished 12 years or more of school education. However, this can be explained by the earlier school enrolment in the UK. The distributions of household size, BMI, total cholesterol, and *APOE* genotypes were comparable between the two cohorts. The prevalence of low-dose ASA use was also similar: 18.3% in ESTHER vs 18.7% in UK Biobank. In logistic regression models adjusted for age and sex, common factors associated with low-dose ASA use in both cohorts were age, male sex, physical inactivity, current smoking, BMI ≥ 30 kg/m^2^, diabetes, hypertension, and CHD (Supplemental Tables A2 and A3).

**Table 1:** Baseline characteristics of included study participants from the ESTHER (*N *= 5258) and UK Biobank study (*N *= 305,394)

Characteristics	ESTHER (***N ***= 5258)	UK Biobank (***N ***= 305,394)
	***n*** **(%)**^**a**^	***n*** **(%)**^**a**^
Age [years]		
55–59	1020 (19.4)	90,134 (29.5)
60–64	1504 (28.6)	120,315 (39.4)
65–69	1577 (30.0)	92,559 (30.3)
70–79	1157 (22.0)	2386 (0.8)
Sex		
Female	2852 (54.2)	163,705 (53.6)
Male	2406 (45.8)	141,689 (46.4)
Low-dose ASA use	962 (18.3)	57,068 (18.7)
Number of individuals in household		
1	854 (16.2)	63,706 (20.9)
2	3244 (61.7)	182,426 (59.7)
>2	1160 (22.1)	59,262 (19.4)
School education [years]		
≤9	3915 (74.5)	91,181 (29.9)
10–11	732 (13.9)	79,493 (26.0)
≥12	611 (11.6)	134,720 (44.1)
BMI [kg/m^2^]		
<25	1409 (26.8)	93,414 (30.6)
25–<30	2543 (48.4)	134,764 (44.1)
≥30	1306 (24.8)	77,216 (25.3)
Smoking		
Never	2757 (52.4)	157,241 (51.5)
Former	1785 (34.0)	120,990 (39.6)
Current	716 (13.6)	27,163 (8.9)
Alcohol consumption ^b^		
None	1615 (30.7)	94,298 (30.9)
Low or moderate	3562 (67.7)	174,310 (57.1)
High	81 (1.5)	36,786 (12.1)
Physical activity ^c^		
Inactive	991 (18.9)	44,280 (18.4)
Low	2439 (46.4)	100,178 (41.7)
Medium or high	1828 (34.8)	95,877 (39.9)
Coronary heart disease		
No	4290 (81.6)	285,316 (93.4)
Yes	968 (18.4)	20,078 (6.6)
Hypertension		
No	2235 (42.5)	202,175 (66.2)
Yes	3023 (57.5)	103,219 (33.8)
Diabetes		
No	4478 (85.2)	286,337 (93.8)
Yes	780 (14.8)	19,057 (6.2)
Depression		
No	4495 (85.5)	273,742 (89.6)
Yes	763 (14.5)	31,652 (10.4)
Total cholesterol [mg/dL]		
<200	1662 (31.6)	97,264 (31.9)
200–<240	1786 (34.0)	102,938 (33.7)
≥240	1810 (34.4)	105,192 (34.4)
HDL [mg/dL]		
<40	968 (18.4)	36,560 (12.0)
40–<60	2614 (49.7)	156,740 (51.3)
≥60	1676 (31.9)	112,094 (36.7)
CRP [mg/L]		
<1	1339 (25.5)	107,846 (35.3)
1–<3	2053 (39.1)	117,782 (38.6)
≥3	1866 (35.5)	79,766 (26.1)
*APOE* genotypes		
ε4 non-carrier	3881 (73.8)	219,088 (71.7)
ε2/ε4 or ε3/ε4	1295 (24.6)	79,134 (25.9)
ε4/ε4	82 (1.6)	7172 (2.4)

*Abbreviations: ASA* acetylsalicylic acid, *BMI* body mass index, *CRP* C-reactive protein, *HDL* high-density lipoprotein

^a^Numbers of imputed complete dataset number 1. The proportion of imputed missing values of each variable is shown in Suppl. Table A2

^b^Definition of low or moderate alcohol consumption: women 0 to 39.99 gram ethanol/day (g/d) or men 0 to 59.99 g/d; definition of high alcohol consumption: women ≥40 to 39.99 g/d or men ≥60 g/d

^c^In ESTHER: “Inactive” was defined by <1 h of vigorous and <1 h light physical activity per week. “Medium or high” was defined by ≥2 h of vigorous and ≥2 h of light physical activity/week. All other amounts of physical activity were grouped into the category “Low”. In UK Biobank: “Inactive” was defined by ≤1 h of performing walking, moderate and vigorous activity. “Medium or high” was defined by >2 h of performing walking, moderate and vigorous activity. All other amounts of physical activity were grouped into the category “Low”

**Fig. 1:**
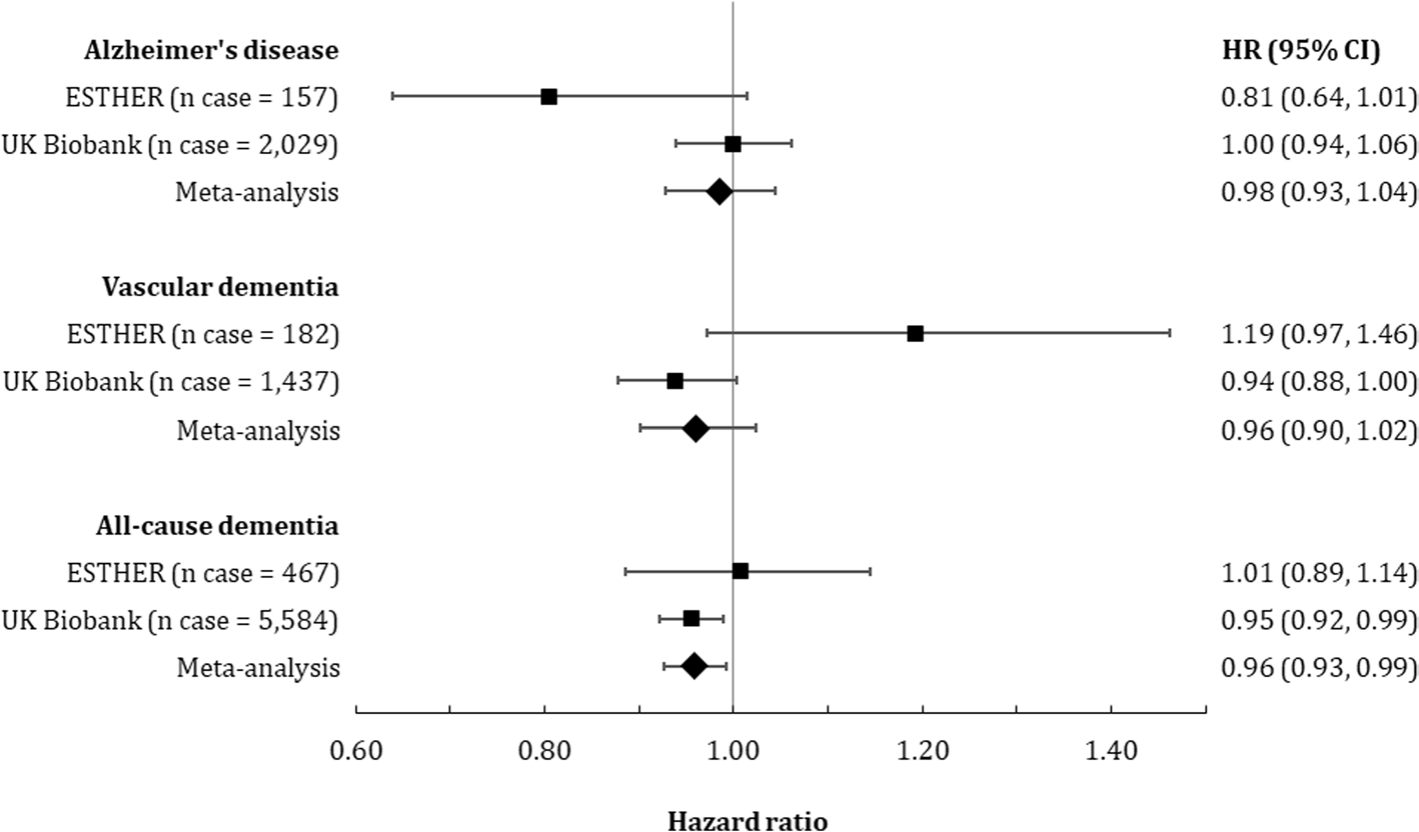
Association between low-dose ASA use and dementia incidence in ESTHER, UK Biobank and the meta-analysis of the two cohorts. The inverse probability of treatment weighting (IPTW) method was used to obtain hazard ratios

Among all included *n *= 5286 participants of the ESTHER study, 476 cases of all-cause dementia were diagnosed during a median follow-up of 14.3 years. Thereof, 157 participants were diagnosed with AD and 182 with VD. Among the included *n *= 305,394 participants from the UK Biobank, 5584 developed all-cause dementia during a median of 11.6 years follow up, of whom 2029 were diagnosed with AD and 1437 with VD. Table 2 shows the longitudinal association between low-dose ASA use and the three dementia outcomes. In the simple model with age, sex, education, *APOE* genotypes, BMI, smoking status, alcohol consumption, physical activity, diabetes, hypertension, CHD, and depression as covariates, no significant associations were found between low-dose ASA use and dementia outcomes for ESTHER. On the other hand, low-dose ASA use was found to be significantly associated with an increased risk of all-cause dementia and VD (HR [95% CI]: 1.12 [1.05 to 1.20] and 1.27 [1.12 to 1.45], respectively) in the UK Biobank cohort. Results did not change much for ESTHER in the main IPTW model but reversed for UK Biobank: low-dose ASA use was significantly associated with a decreased risk of all-cause dementia (HR [95% CI]: 0.95 [0.92 to 0.99]). When excluding dementia cases that were diagnosed during the first 5 years of follow-up, an inverse association of low-dose ASA and AD also became statistically significant in ESTHER (HR [95% CI] 0.68 [0.51 to 0.89]). However, results for the UK Biobank did not change much. Meta-analysis of the IPTW model results of both cohorts resulted in weak, inverse associations of low-dose ASA with all dementia outcomes, of which only the pooled effect estimate for all-cause dementia was statistically significant (HR [95% CI]: 0.96 [0.93 to 0.99] (Fig. 1).

**Table 2:** Longitudinal association of low-dose ASA use with all-cause and common subtype dementia incidence

	ESTHER, ***N ***= 5286						UK Biobank, *N* = 305,394					
	All-cause dementia		Alzheimer’s disease		Vascular dementia		All-cause dementia		Alzheimer’s disease		Vascular dementia	
	*n*_case_	HR (95% CI)	***n***_**case**_	HR (95% CI)	***n***_**case**_	HR (95% CI)	***n***_**case**_	HR (95% CI)	***n***_**case**_	HR (95% CI)	***n***_**case**_	HR (95% CI)
Simple model^a^	476	0.93 (0.73, 1.18)	157	0.68 (0.43, 1.07)	182	1.00 (0.69, 1.45)	5,584	**1.12 (1.05, 1.20)**	2029	1.11 (0.99, 1.25)	1437	**1.27 (1.12, 1.45)**
IPTW model	476	1.01 (0.89, 1.14)	157	0.81 (0.64, 1.01)	182	1.19 (0.97, 1.46)	5,584	**0.95 (0.92, 0.99)**	2029	1.00 (0.94, 1.06)	1437	0.94 (0.88, 1.00)
IPTW model plus 5-year lag-time^b^	409	0.93 (0.81, 1.08)	129	**0.68 (0.51, 0.89)**	158	1.23 (0.99, 1.53)	4,855	**0.94 (0.90, 0.97)**	1744	1.00 (0.94, 1.07)	1225	0.95 (0.88, 1.02)

Note: Statistically significant results are printed in bold

*Abbreviations: IPTW*, inverse probability of treatment weighting

^a^Simple model was adjusted for age, sex, education, APOE genotypes, BMI, smoking status, alcohol consumption, physical activity, diabetes, hypertension, coronary heart disease, and depression

^b^Dementia cases, which happened within the first 5 years of follow-up were excluded, resulting in a total sample size of *N*=5,191

**Fig. 2:**
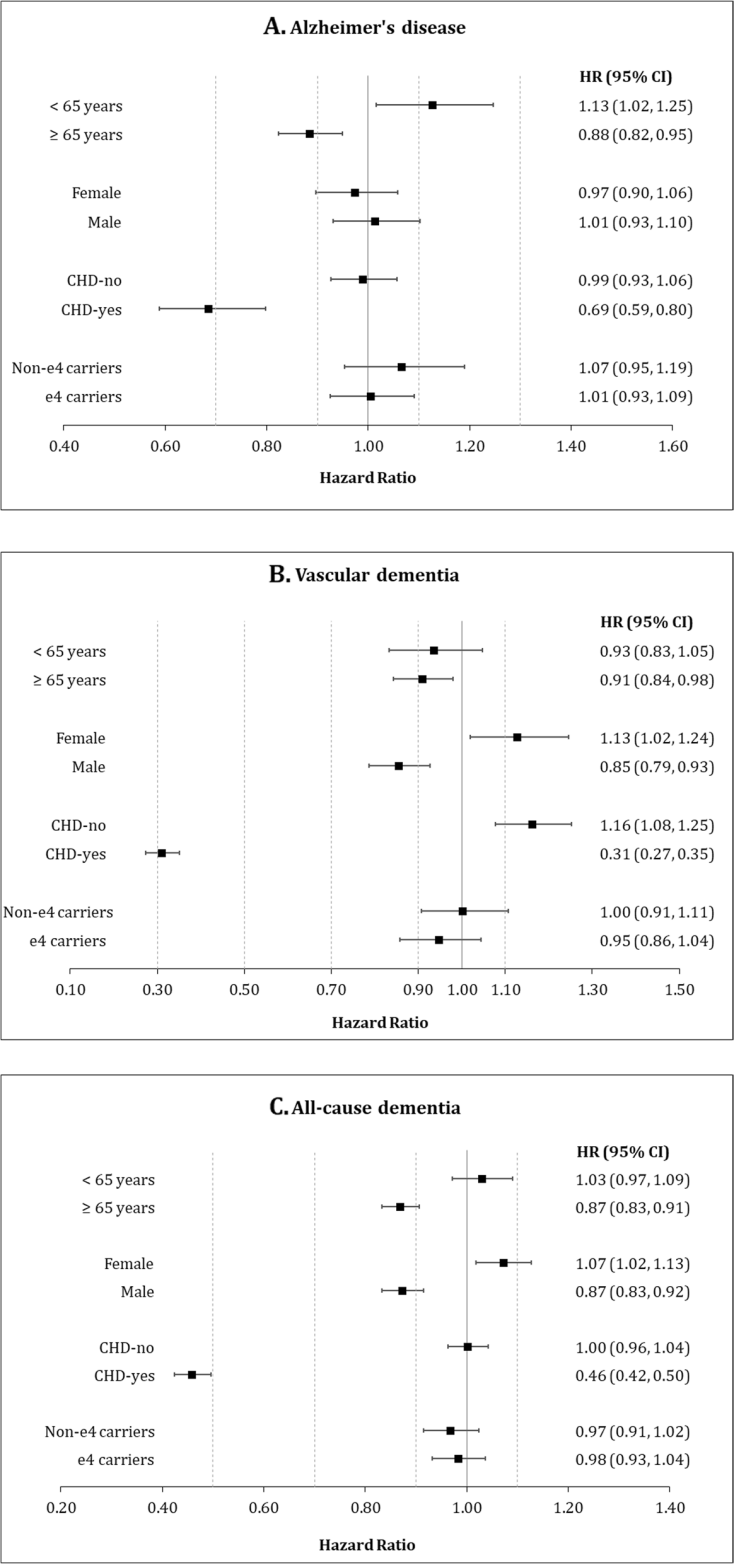
Meta-analysis of the association between low-dose ASA use and dementia outcomes, stratified by age, sex, CHD, and *APOE* ε4-carrier status. The inverse probability of treatment weighting (IPTW) method was used to obtain hazard ratios. **A** Alzheimer’s disease. **B** Vascular dementia. **C** All-cause dementia

Results of the main IPTW model stratified by age, sex, CHD, and *APOE* genotype are shown in Supplemental Table A5 and Fig. 2. In the meta-analysis, low-dose ASA use was associated with a decreased hazard for all dementia outcomes in participants aged ≥65 years. Furthermore, low-dose ASA use was associated with decreased risk for all-cause dementia and VD incidence in males and not in females. The results did not differ between *APOE ε4* carriers and non-carriers. The strongest protective association of low-dose ASA use was observed in participants with CHD. In particular, low-dose ASA use was associated with 31%, 69%, and 54% reduced the risk of developing AD, VD, and all-cause dementia, respectively (Fig. 2). A statistically significant interaction between CHD and low-dose ASA use was observed for all three outcomes in both cohorts (Supplemental Fig. A3). The meta-analyzed p-values for the interaction terms were all <0.001 for all-cause dementia, VD, and AD. We also conducted analyses stratified further by both age and CHD (Supplemental Table A6) and by both sex and CHD (Supplemental Table A7). These analyses revealed that CHD was the main effect-modifier in the association between low-dose ASA and dementia outcomes, while age and sex played no important role.

We included *N *= 136,589 participants aged 55 years and older in the sensitivity analysis on different durations of low-dose ASA use (Table 3). Of those, 100,252 (73.5%) had no low-dose ASA prescription at baseline. Two-thirds of low-dose ASA users (*N *= 24,200, 66.6%) had their first low-dose ASA prescription more than 10 years prior to end of follow-up and 36.8% of these long-term users of low-dose ASA had been diagnosed with CHD. Compared to non-users, those long-term users had approximately half the risk for the three dementia outcomes of non-low-dose ASA users (HR point estimates between 0.48 and 0.58), while users of 5 to 10 years had no decreased risk.

**Table 3:** Association between different duration of low-dose ASA use prior to end of follow-up identified by primary care data (UK Biobank) with all-cause and common subtype dementia incidence (*N *= 136,589)

	***N*** total	Prevalence of CHD (%)	All-cause dementia		Alzheimer’s disease		Vascular dementia	
			***n***_**case**_	OR (95% CI) ^**a**^	***n***_**case**_	OR (95% CI) ^**a**^	***n***_**case**_	OR (95% CI) ^**a**^
Never-user	100,252	2.7	1427	Ref	521	Ref	318	Ref
User for ≤5 years prior to end of follow-up	2385	32.8	216	**Excluded** ^**b**^	64	**Excluded** ^**b**^	99	**Excluded** ^**b**^
User for >5 to ≤10 years prior to end of follow-up	9752	36.1	309	0.95 (0.86, 1.05)	110	1.02 (0.86, 1.20)	92	0.87 (0.73, 1.03)
User for > 10 years prior to end of follow-up	24,200	36.8	595	**0.51 (0.47, 0.56)**	198	**0.58 (0.51, 0.68)**	197	**0.48 (0.42, 0.56)**

Note: Statistically significant results are printed in bold

^a^Results of multivariable logistic regression models, adjusted for covariates related to cardiovascular risk (all variables shown in Supplementary Table A3)

^b^Excluded due to protopathic bias (see Discussion, Strengths and Limitations of this Study)

## Discussion

In this individual participant data meta-analysis of two large cohort studies, the use of low-dose ASA was weakly associated with decreased all-cause dementia incidence but not with AD and VD incidence. However, after stratifying by CHD, it became apparent that only subjects with pre-existing CHD benefited strongly from low-dose ASA use. The results were concordant across both cohorts and validated by significant interaction terms of CHD and low-dose ASA use observed in both cohorts. In addition, when participants were compared based on the length of low-dose ASA use, a strong protective effect was only observed among low-dose ASA users, who started the use 10 years or more prior to end of follow-up. In the latter analysis, 36.8% of users of low-dose ASA for 10 or more years were CHD patients, but we assume that this number is underestimated in the primary care data of the UK Biobank and that all of these long-term low-dose ASA users use it for cardiovascular risk prevention and have CHD or a diagnosis of a related cardiovascular disease. Thus, we do not think that this result contradicts the previous finding that the effectiveness of low-dose ASA strongly depends on pre-existing CHD and that other persons from the general population had no decreased risk of dementia if they were low-dose ASA users.

### Biological mechanisms

Several suggested mechanisms could explain a potential protective effect of ASA use on both AD and VD development. The primary pharmacological activity of ASA is the inhibition of the cyclooxygenase (COX) enzymes, leading to a reduction in the levels of prostaglandins, prostacyclin, and thromboxanes. Those are important in AD pathogenesis [3, 5], and in the prevention of ischemic brain damage [29], a strong risk factor for VD [6]. Along with the thromboxane pathway, low-dose ASA also inhibits platelet activation and aggregation, which can prevent transient cerebral ischemic attacks and eventually help to enhance the blood flow in the cognitive area [29, 30].

ASA could further reduce the related pathology effect on AD, primarily by reducing amyloid-beta (Aβ). Firstly, it activates the peroxisome-proliferator-activated receptor-γ (PPARγ), which controls the expression of pro-inflammatory genes [2], downregulates beta-secretase 1 (BACE1), and thereby reduces amyloid precursor protein (APP) cleavage and Aβ production [4]. However, it is possible that the 31% reduction in hazard for AD is restricted to those participants with mixed dementia due to the coexistence of AD and VD and thus mediated through a reduction in hazard of the VD component of AD. What speaks in favor of this hypothesis is that neuropathological studies suggest that this type of mixed dementia is a rather common pathological finding in the elderly with a prevalence of about 22% [31].

Cardio- and cerebrovascular disease and dementia often not only coexist and pose risks for each other; in addition, it is well-known that vascular and neurodegenerative pathologies could interact additionally and synergistically [32]. Imaging studies have demonstrated that vascular risk factors may contribute to Aβ deposition in the brain [33, 34]. Incident CHD has previously been found to be associated with accelerated long-term cognitive decline [35, 36]. Furthermore, people with pre-existing CHD have an increased risk for recurrent vascular events than CHD-free individuals [37, 38]. Therefore, there are plausible mechanisms in favor of the hypothesis that low-dose ASA is more effective in preventing dementia among CHD patients than in the CHD-free population. Support for this hypothesis can also be drawn from the study of Kern et al. [39], which observed that women with high risk of cardiovascular disease (CVD) (Framingham risk score of more than 10%) who used low-dose ASA had a decreased loss of cognitive function over a follow-up of 5 years compared to women who did not use low-dose ASA (Mini-Mental State Examination (MMSE) score change − 0.33 vs − 0.95; *p *= 0.028).

### Comparison with other observational studies

Very few longitudinal studies investigated the associations of low-dose ASA use with dementia incidence. None of the existing was population-based, had specifically evaluated the importance of the duration of low-dose ASA use, had VD as an outcome, or tested a potential interaction of ASA use and CHD. A systematic review by H. Li et al. summarized the literature up to April 2020 and we checked in PubMed that there were no further studies on low-dose ASA use and dementia outcomes as of August 2021 [7]. Overall, a meta-analysis of 8 studies indicated that the use of any dose of ASA did not significantly decrease the risk of developing dementia (pooled relative risk (RR) [95% CI]: 0.94 [0.77 to 1.16]. However, when the authors restricted the meta-analysis to 4 studies with low-dose ASA exposure and the outcome all-cause dementia [39–42] and 2 studies with low-dose ASA exposure and the outcome AD [40, 41], low-dose ASA use showed a protective effect against all-cause dementia (pooled RR [95% CI]: 0.82 [0.71 to 0.96]) and AD (pooled RR [95% CI]: 0.54 [0.33 to 0.89]). Among the four studies on all-cause dementia, three reported statistically significant results, and only the study of Kern et al. [39], which was underpowered (*n* = 41 cases), showed no statistical differences between low-dose ASA users and non-users regarding the 5-year risk of dementia. None of the 4 individual studies is comparable to our study populations from the UK Biobank and ESTHER (general population, age ≥55) because they included either only women [39], twins aged 80 years or older [41], patients with late-onset depression [42] or type 2 diabetes patients [40]*.*

A systematic review of cohort studies on the association of NSAIDs use and AD incidence was conducted by C. Zhang et al. with literature search up to April 2017 [43]. In this review, 16 cohorts with a total of 236,022 participants could be included in the meta-analysis, and ever use of NSAIDs was also found to be statistically, significantly associated with a reduced risk of AD (RR [95%CI], 0.81 [0.70 to 0.94] [43]. A further meta-analysis limited to 10 studies with ASA (any dose) as exposure yielded a similar RR but without statistical significance (RR [95%CI], 0.89 [0.70 to 1.13]).

In summary, previous observational studies showed some hints that the anti-inflammatory actions of NSAIDs could prevent AD. However, the strongest and most consistent results were observed in low-dose ASA, likely because they are the only group of NSAIDs used mainly long-term for CVD prevention but not occasionally for the indication of pain.

### Findings from randomized controlled trials

Up to date, only two RCTs on the topic were published (both in 2020) and the meta-analysis of their results was a null result [7]. In the Japanese Primary Prevention of Atherosclerosis With Aspirin for Diabetes (JPAD) trial [44], 2536 diabetes patients without CVD (age 30–85 years; median: 65 years) were randomized into receiving low-dose ASA (81 or 100 mg) for a median of 4.4 years and were followed up over a median of 11.4 years. A tendency towards reducing all-cause dementia risk was observed, but the result was not statistically significant (HR [95%CI]: 0.82 [0.58 to 1.16]). When stratified by sex, however, women in the low-dose ASA group had a lower incidence of dementia compared with those in the non-ASA group (HR [95%CI]: 0.47 [0.25 to 0.86]). The Aspirin in Reducing Events in the Elderly (ASPREE) trial [45] followed 19,114 community-dwelling older adults from the US and Australia without CVD and physical disability, aged 65–98 years over a median of 4.7 years. The authors observed that there was no difference in the incidence of AD between the low-dose ASA group (100 mg) and the placebo group (HR [95%CI] for clinically probable AD: 0.98 [0.83 to 1.15]) and for clinically possible AD: 1.03 (0.83-1.27).

Our results indicate that a protective effect of low-dose ASA on dementia incidence could only be found among CHD patients. This possibly explains the obtained null results in the main analysis of the JPAD and ASPREE trials in which patients with CVD were excluded. Furthermore, as the average age of the participants was relatively high, the initiation of low-dose ASA use may have been already too late. Previous research has proposed that the pathological changes of dementia could start more than two decades before the onset of clinical symptoms. In addition, ASA was shown not to affect the cognitive decline in individuals already diagnosed with dementia [46, 47]. In our data, low-dose ASA only showed a protective effect if participants had taken it for at least 10 years, as these people started at a relatively young age and took low-dose ASA sufficiently long. The 4.7-year treatment period in the ASPREE trial [45] was presumably too short and could be the reason for the null result. The treatment period in the JPAD trial [44] was also only for 4.4 years but they had an extended observational follow-up of 11.4 years, in which 84% of the study participants retained their medication. This might explain, why the JPAD trial had an effect estimate that tended towards a protective effect whereas the ASPREE trial had not. It will be of utmost interest, whether the results of the APREE trial change when an extended observational follow-up of at least 10 years has been completed.

### Public health implications

Potential recommendations for the primary prevention of dementia that could follow our analysis need to be made in context with the existing low-dose ASA guidelines for primary CVD and CRC prevention. A relatively high cardiovascular risk, the initiation of low-dose ASA use at middle age and the willingness of the patient to take low-dose ASA for at least 10 years are the known essential factors for a favorable benefit-risk ratio of low-dose ASA in guidelines for primary prevention of both CVD and colorectal cancer (CRC) [48–61].

A comprehensive decision analysis has concluded that lifetime low-dose ASA use initiated at middle age (40 to 69 years) in persons with higher CVD risk has a favorable benefit-risk ratio in the primary prevention of CVD and colorectal cancer (i.e., outweighs its hemorrhage risks) [60]. Based on this analysis, the United States Preventive Services Task Force (USPSTF) recommends the preventive use of low-dose ASA for all adults aged 40–59 years with a 10-year risk of a CVD event greater than 10% and a willingness to take low-dose ASA daily for at least 10 years [61]. Even though the USPSTF guideline targets CVD and CRC prevention, based on our results, following this recommendation could also be an effective measure for the prevention of dementia. Currently, the evidence from RCTs is too weak to include dementia as a factor in decision analyses on low-dose ASA use. However, this might change in the future when more long-term RCTs targeting the right population (middle age and relatively high CVD risk) are published.

### Strengths and limitations of this study

The strengths of this study include the prospective cohort design, the large sample size (*n *= 5258 for ESTHER, *n *= 305,394 for UK Biobank), and a long follow-up period (median of 14.3 for ESTHER and 11.6 years for UK Biobank). In addition, evaluating the association of different durations of low-dose ASA use was possible due to utilizing the primary care data. This analysis is not biased by the issue that older study participants are more likely to have both longer exposure probability towards low-dose ASA and a higher chance to be diagnosed with dementia during follow-up because first, the analysis was adjusted for age, and second, it is plausible that even some study participants with the minimum age included in our cohorts of 55 years have used low-dose ASA for more than 10 years because of some start with this therapy in their 40s in adherence with the current guideline for CVD prevention [60, 61].

This study also has some limitations. As with any observational study, residual confounding remains possible, and causation cannot be tested like in RCTs. However, by applying the inverse probability of treatment weighting using a propensity score including all main cardiovascular risk factors, we were able to balance the distribution of CVD risk factors between low-dose ASA users and non-users, and thus, were able to adjust for confounding by indication comprehensively.

There are limitations with respect to the exposure assessment during the follow-up. In the UK Biobank, low-dose ASA use was ascertained via linked primary care data, which could be incomplete. In the ESTHER study, low-dose ASA use was ascertained via questionnaires filled by study participants and their GPs every 3 years. While there is a high completeness at these follow-up time points, no information is available about the time in between. In addition, drug adherence was not assessed in both studies. However, both cohort studies had excellent assessments of low-dose ASA use at baseline, which included not only prescribed but also low-dose ASA bought over-the-counter.

Regarding dementia outcome assessment, the main limitation of the use of linkage to electronic health records in the UK Biobank, with most cases originating from hospital records, is that milder cases of dementia diagnosed in the outpatient setting could have been missed [62]. The dementia ascertainment in the ESTHER study likely includes more milder dementia cases due to collecting medical records from specialists via participant’s GPs. However, there was no study protocol for specific dementia diagnostics needed to follow to obtain these diagnoses. The applied procedures in the UK Biobank and ESTHER study reflect the current routine of diagnosing dementia by British and German clinicians, which is a strength of this study design because it enhances the generalizability of the results. This can also explain the low proportion of diagnosed AD among the all-cause dementia cases. Many dementia cases have a missing specific diagnosis simply because differential diagnostics are often not made in routine practice in the community setting.

Protopathic bias was present in the analysis of the primary care data of the UK Biobank with more than 2-fold increased odds ratios for dementia outcomes of low-dose ASA users who initiated low-dose ASA use less than 5 years prior to dementia diagnosis. Low-dose ASA use often gets initiated after CVD events and these patients have an increased risk of recurrent CVD events. As most of the dementia diagnoses in the UK Biobank originate from hospital records, an accumulation of first-time dementia diagnoses in the data set among patients hospitalized for recurrent CVD events that happen up to 5 years after low-dose ASA initiation must be expected, since these hospitalizations are needed for awareness of dementia diagnoses in the UK Biobank. Therefore, a sufficient lag time of 5 years between low-dose ASA initiation and dementia diagnosis needs to be ascertained in this analysis by excluding patients with short-term low-dose ASA use.

## Conclusions

In conclusion, in this analysis of two large, population-based cohort studies from Germany and the UK, low-dose ASA demonstrated a protective potential for AD, VD, and all-cause dementia among study participants with pre-existing CHD, but not in other persons from the general population. Furthermore, taking the drug for more than 10 years was critical for detecting the association. This implies that people with CHD may not only profit from long-term low-dose ASA use by reducing their CVD risk but also their dementia risk. The results of this study can only be generalized to mainly Caucasian populations aged 55 years and older, and the findings need to be further tested by RCTs with large sample sizes and long follow-up periods.

## Supplementary Information

**Additional file 1.**

## Data Availability

Data from ESTHER is available upon reasonable request that is compatible with participants’ informed consent. Data from the UK Biobank (https://www.ukbiobank.ac.uk/) is available to bona fide researchers on application.

## Abbreviations

Aβ: Amyloid-beta
AD: Alzheimer’s disease
APOE: Apolipoprotein E
APP: Amyloid precursor protein
ASA: Acetlysalicylic acid
ASCVD: Atherosclerotic cardiovascular disease
BACE1: Beta-secretase 1
BMI: Body mass index
BNF: British National Formulary
CHD: Coronary heart disease
CI: Confidence interval
COX: Cyclooxygenase
CRC: Colorectal cancer
CRP: C-reactive protein
CVD: Cardiovascular disease
ESTHER: Epidemiologische Studie zu Chancen der Verhütung, Früherkennung und optimierten Therapie chronischer Erkrankungen in der älteren Bevölkerung
GP: General practitioner
HDL: High-density lipoprotein
VD: Vascular dementia
HR: Hazard ratio
IPTW: Inverse probability of treatment weighting
low-dose ASA: Low-dose acetylsalicylic acid
MMSE: Mini-Mental State Examination
NSAID: Non-steroidal anti-inflammatory drug
PPAR: Peroxisome-proliferator-activated receptor
RCTs: Randomized controlled trials
RR: Relative risk
PS: Propensity score
UK: United Kingdom
USPSTF: United States Preventive Services Task Force
